# Coronavirus Disease 2019 (COVID-19) in Italy: Double Reading of Chest CT Examination

**DOI:** 10.3390/biology10020089

**Published:** 2021-01-25

**Authors:** Alfonso Reginelli, Roberta Grassi, Beatrice Feragalli, Maria Paola Belfiore, Alessandro Montanelli, Gianluigi Patelli, Michelearcangelo La Porta, Fabrizio Urraro, Roberta Fusco, Vincenza Granata, Antonella Petrillo, Giuliana Giacobbe, Gaetano Maria Russo, Palmino Sacco, Roberto Grassi, Salvatore Cappabianca

**Affiliations:** 1 Department of Precision Medicine, Università degli Studi della Campania Luigi Vanvitelli, 80121 Naples, Italy; alfonso.reginelli@unicampania.it (A.R.); roberta.grassi89@gmail.com (R.G.); mariapaola.belfiore@unicampania.it (M.P.B.); fabrizio.urraro@unicampania.it (F.U.); giuliana.giacobbe@unicampania.it (G.G.); gaetanomaria.russo@unicampania.it (G.M.R.); roberto.grassi@unicampania.it (R.G.); salvatore.cappabianca@unicampania.it (S.C.); 2 Oral and Biotechnological Sciences—Radiology Unit “G. D’Annunzio”, Department of Medical, University of Chieti-Pescara, 66100 Chieti, Italy; beatriceferagalli@hotmail.com; 3 Laboratory Medicine Unit, ASST Bergamo Est, 24068 Seriate, Italy; al.montanelli@asst-bergamoest.it; 4 Department of Radiology, ASST Bergamo Est, 24068 Seriate, Italy; patellig@yahoo.it; 5 Department of Radiology, UOC San Severo Hospital, 71016 San Severo, Italy; m.laporta.neuro@gmail.com; 6 Radiology Division, Istituto Nazionale Tumori IRCCS Fondazione Pascale—IRCCS di Napoli, 80131 Naples, Italy; v.granata@istitutotumori.na.it (V.G.); a.petrillo@istitutotumori.na.it (A.P.); 7 Diagnostic Imaging Unit, Department of Radiological Sciences, Azienda Ospedaliera Universitaria Senese, 53100 Siena, Italy; saccopal@gmail.com; 8 Foundation SIRM, 20122 Milan, Italy

**Keywords:** COVID-19, chest CT, structured report, double reading, radiological signs

## Abstract

**Simple Summary:**

The objective of the manuscript was to assess the performance of the second reading of chest compute tomography (CT) examinations by expert radiologists in patients with discordance between the reverse transcription real-time fluorescence polymerase chain reaction (RT-PCR) test for COVID-19 viral pneumonia and the CT report. Double reading of CT could increase the diagnostic confidence of radiological interpretation in COVID-19 patients in a pandemic area. Using second readers and a structured report for COVID-19 diagnosis could reduce the rate of discrepant cases between RT-PCR result and CT diagnosis for COVID-19 viral pneumonia.

**Abstract:**

To assess the performance of the second reading of chest compute tomography (CT) examinations by expert radiologists in patients with discordance between the reverse transcription real-time fluorescence polymerase chain reaction (RT-PCR) test for COVID-19 viral pneumonia and the CT report. Three hundred and seventy-eight patients were included in this retrospective study (121 women and 257 men; 71 years median age, with a range of 29–93 years) and subjected to RT-PCR tests for suspicious COVID-19 infection. All patients were subjected to CT examination in order to evaluate the pulmonary disease involvement by COVID-19. CT images were reviewed first by two radiologists who identified COVID-19 typical CT patterns and then reanalyzed by another two radiologists using a CT structured report for COVID-19 diagnosis. Weighted k values were used to evaluate the inter-reader agreement. The median temporal window between RT-PCRs execution and CT scan was zero days with a range of (−9, 11) days. The RT-PCR test was positive in 328/378 (86.8%). Discordance between RT-PCR and CT findings for viral pneumonia was revealed in 60 cases. The second reading changed the CT diagnosis in 16/60 (26.7%) cases contributing to an increase the concordance with the RT-PCR. Among these 60 cases, eight were false negative with positive RT-PCR, and 36 were false positive with negative RT-PCR. Sensitivity, specificity, positive predictive value and negative predictive value of CT were respectively of 97.3%, 53.8%, 89.0%, and 88.4%. Double reading of CT scans and expert second readers could increase the diagnostic confidence of radiological interpretation in COVID-19 patients.

## 1. Introduction

Coronavirus disease (COVID-19) is an infectious disease caused by a newly discovered coronavirus. Coronaviruses are a large family of viruses transmitting between animals and people that cause illnesses ranging from the common cold to more severe diseases such as Middle East respiratory syndrome (MERS-CoV) and severe acute respira11tory syndrome (SARS-CoV). COVID-19 continues its spread throughout the world [1,2]. 

The COVID-19 diagnosis is established using a reverse transcription real-time fluorescence polymerase chain reaction (RT-PCR) test. RT-PCR assays can be performed on nasopharyngeal and/or oropharyngeal swabs, sputum, blood samples, body fluids, stool samples, and bronchoalveolar lavage fluid using qualitative and quantitative approaches [3]. In the literature, it is reported that qualitative RT-PCR has low sensitivity due to a high number of false negatives (about 20%) [4,5]. However, the RT-PCR test is the most reliable test for COVID-19 infection—several studies have also reported a high false positive rate of this test and a sensitivity of 60–70% [5]. In addition, quantitative RT-PCR is different from qualitative RT-PCR. It was also demonstrated by Poon et al., 2004 [4], that the quantitative real-time RT-PCR test was more sensitive than conventional qualitative RT-PCR in SARS-CoV-2 detection using samples collected early in the disease course. On days one to three, the quantitative RT-PCR test was able to detect SARS-CoV-2 in one-half of nasopharyngeal samples while qualitative conventional RT-PCR only one-third. At days 7–10, the detection rates of the quantitative and qualitative RT-PCR test had comparable findings. This indicated that quantitative RT-PCR tests are a better diagnostic method for early SARS diagnosis in respect to the qualitative conventional RT-PCR assay. Therefore, quantitative RT-PCR could be gainfully employed for risk stratification for the detection of asymptomatic patients to reduce the risk of contagious [6].

Recent results have revealed the efficiency of some imaging methods, including chest radiographs (X-rays) and chest computed tomography (CT) scans, in the management of COVID-19 disease. Both chest X-ray and CT scans can evaluate the pulmonary involvement by an abnormality that could be linked to the COVID-19 infection but also at other infections. In fact, several radiological societies do not recommend chest X-ray or CT for the screening or diagnosis of COVID-19 [7,8,9,10]. 

Chest X-ray examination, although not offering highly specific findings, provides a first overview of the patients, especially in the emergency room, and can direct the differential diagnosis towards other infections that determine pulmonary parenchymal involvement, other than COVID-19 infection [11]. The typical radiological pattern on chest X-rays was patchy or diffuse asymmetric airspace opacities. [11,12]. Radiologists cannot make a safe diagnosis of COVID-19 disease based on a chest X-ray alone. Bandirali et al. [12], in their study on 170 patients, demonstrated that in 100/170 X-rays there were pulmonary abnormalities highly suspicious for COVID-19 pneumonia. 

CT examination was used to evaluate the grade and the extent of the viral pneumonia by COVID-19 [13,14]. Radiologists focus on main CT findings (ground-glass opacity, consolidation) and lesion distribution (bilateral, multilobar). Bilateral distribution of ground-glass opacities (GGOs), with or without consolidation, in peripheral lungs was reported as a characteristic feature of COVID-19 [13,14,15]. However, CT scans can share some similar imaging features between COVID-19 and other types of pneumonia, making differentiation difficult [16,17,18,19,20,21,22]. Moreover, a double reading and interpretation of CT images could have discordant results [22,23,24,25,26,27]. The aim of this study was to assess the performance of the second reading of chest CT using a structured report in patients with discordance between an RT-PCR test and the first CT diagnosis for COVID-19 viral infection.

## 2. Materials and Methods

### 2.1. Patient Characteristics

“Bergamo Est” Institutional Review Institute (IRB) approved the study and renounced the written informed consent for this retrospective study, considering the ongoing epidemic emergency, which assessed the unidentified data and did not involve potential risks for patients. 

Three hundred and seventy-eight patients were included in this retrospective study (121 women and 257 men; 71 years median age, with a range of 29–93 years) subjected to a qualitative RT-PCR test for suspicious COVID-19 infection, between 23 February 2020 and 5 March 2020. The virus investigation for COVID-19 diagnosis was performed by the current gold standard test in the clinical laboratory of ASST Bergamo Est (Seriate, Italy).

Table 1 illustrates demographic characteristics and CT findings and performance in the detection of pneumonia infection by Coronavirus Disease (COVID-19) in patients with confirmed COVID-19 upon an RT-PCR test.

### 2.2. CT Technique and Analysis

A chest CT scan was performed at the time of patient admission in the hospital using two CT scanners (CT 128 slice Ingenuity of Philips, Amsterdam-Netherlands, and CT 128 slice Optima 660 of GE Healthcare, Chicago, IL, USA). Table 2 reports the chest CT protocol parameters for both scanners. 

Every chest CT examination was evaluated first by two double-blind radiologists with 10- and 7-years’ experience of chest CT (A.M. and G.P. in the midst of the pandemic), respectively. Radiologists observed, blinded to RT-PCR results, the main CT findings suggestive for COVID-19 disease: localization and distribution of GGO and consolidations, crazy paving pattern, and presence of nodules.

RT-PCR results were compared to the CT reports, and other two radiologists blinded each other (B.F. and A.R.) reanalyzed the cases with discordance between CT diagnosis and RT-PCT test, using a structured report for COVID-19 disease defined by the Italian Society of Medical Radiology and Interventional Radiology (SIRM, Milan, Italy) in collaboration with the Exprivia Healthcare company (Bari, Italy) [23].

The structured report includes, for the radiological signs section, a targeted, systematic, and comprehensive description of all abnormalities and a description of the features that are relevant to the suspected pathology. Main CT features included in the report are the extension, distribution, and localization of GGO and consolidations, air bronchogram sign, septal thickening, crazy paving pattern, “reversed halo” sign, nodules, pleural effusion, pericardium effusion, presence of mediastinal lymphadenopathy, the diameter of the main pulmonary artery (more or less than 29 mm) and of the segmental arterial vessels, and barotrauma sign [24,25]. In the second reading, the radiologists, in addition to reviewing the CT, expressed a diagnostic confidence rating on a scale of one to three (low, medium, high). Table 2 reports the CT findings considered for assignment of the diagnostic confidence level.

### 2.3. Statistical Analysis

Continuous data were expressed with median value and range while categorical ones are reported as counts and percentages. The Mann–Whitney and Chi-square tests were used to verify differences respectively between groups of continuous variables and between percentage values among groups.

Weighted k values were used to evaluate the inter-reader agreement. k coefficients in the range of 0.81–1.0 indicated excellent agreement; those in the range of 0.61–0.80, substantial agreement; those in the range of 0.41–0.60, moderate agreement; those in the range of 0.21–0.40, fair agreement; those in the range of 0.00–0.20, poor agreement.

*p*-value < 0.05 was considered significant. Statistical analysis was effected using the Statistics Toolbox of Matlab R2007a (The Math-Works Inc., Natick, MA, USA).

## 3. Results

The median temporal window between RT-PCRs execution and CT scan was zero days with a range of (–9, 11) days. A total of 35/378 (9.3%) patients repeated the RT-PCR test: among these 35 cases, 29/35 (82.9%) resulted negative at the first RT-PCR test were then resulted positive at the second test.

Inter-reader agreement of CT diagnosis attributed by the two radiologists was substantial k = 0.8.

**Figure 1:**
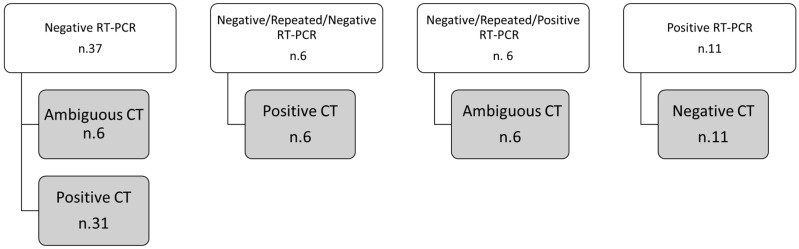
Schematic diagram of patients with discordant findings between RT-PCR test and CT.

Discordance between RT-PCR test and CT findings was revealed in 60 cases (see Figure 1). The discordant cases were prevalently negative at RT-PCR 43/60 (71.7%). Of these 60 cases, a second RT-PCR test was required for 12 (20%) patients. We are inclined to consider unqualified sampling or low viral load in the early stages were responsible for the negative discovery of RT-PCR. In fact, 82.9% of repeated RT-PCR tests ended in a positive finding.

The second CT reading, using the structured report, changed the CT diagnosis in 16/60 (26.7%) cases contributing to an increase in the concordance with the RT-PCR test: seven cases resulted negative for viral pneumonia while nine cases resulted positive for viral pneumonia. Among these 60 cases, 15 had negative CT diagnosis for COVID-19, while 45 had a positive diagnosis at CT for viral pneumonia: 8/15 were false negatives with positive RT-PCR, and 36/45 were false positives with negative RT-PCR. 

Sensitivity, specificity, positive predictive value, and negative predictive value of CT at the first reading were respectively of 94.3%, 55.1%, 89.0%, and 71.7%. 

Sensitivity, specificity, positive predictive value, and negative predictive value of CT were respectively of 97.3%, 53.8%, 89.0%, and 88.4%.

The diagnostic confidence grade (CDG) in the 45 cases with suspicious CT for COVID-19 infection was in 14/45 (31.1%) equal to two and in 31/45 (68.9%) equal to three. The diagnostic confidence grade (CDG) in the 15 cases with negative CT diagnosis for COVID-19 was in 4/15 (26.7%) equal to one while for the remaining 14 cases the described alterations were considered indicative of other diagnoses.

Inter-reader agreement of diagnostic confidence between the radiologists ranged from substantial to excellent (k range, 0.66–0.94).

**Figure 2:**
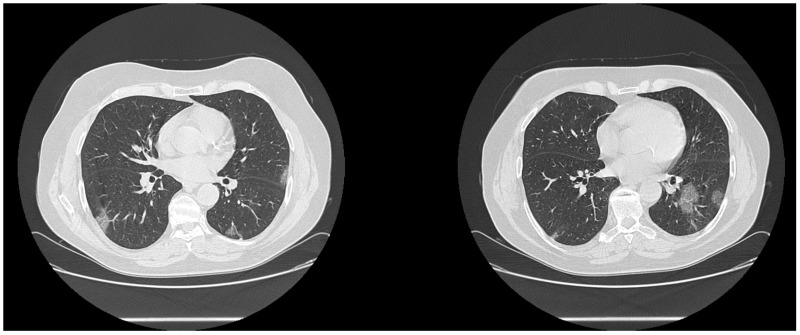
CT examination of a patient with clinical suspicion of COVID-19 infection and negative RT-PCR test. The CT images show the presence of GGO areas with typical roundish morphology and a predominantly peripheral distribution, a suspect picture for COVID-19 infection. The second RT-PCR test showed positive results.

**Figure 3:**
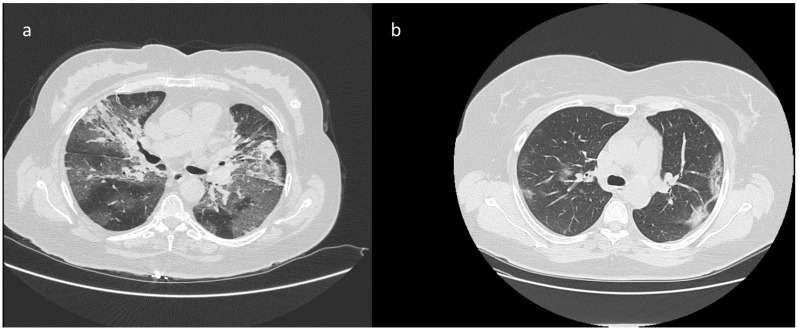
Patients with a positive RT-PCR test for COVID-19 infection. The CT exams detected areas of GGO and parenchymal consolidation with multifocal diffuse (**a**) or predominantly peripheral distribution (**b**).

**Figure 4:**
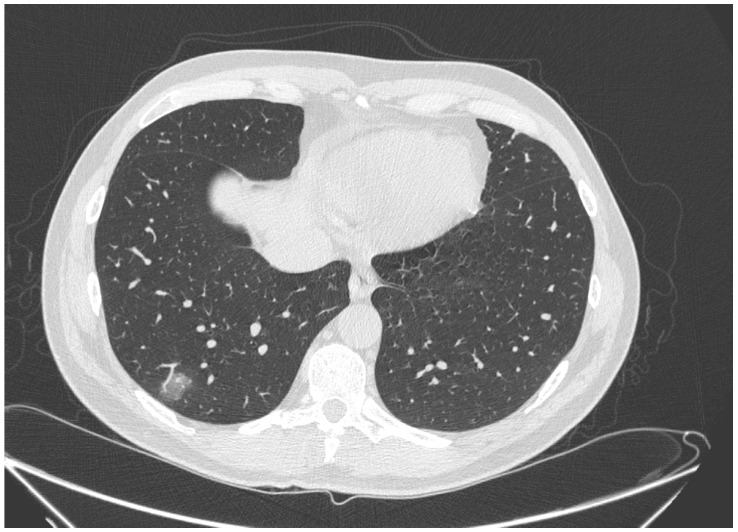
CT examination of a patient with a positive RT-PCR test for COVID-19 infection. The same CT shows a single localization of GGO in the right lower lobe with a rounded morphology.

**Figure 5:**
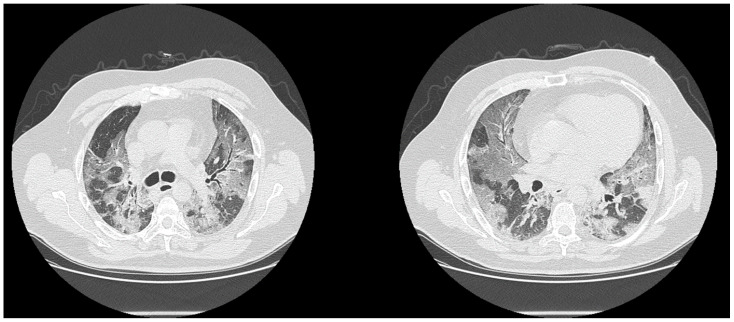
The CT images show a typical aspect of bilateral extended crazy paving characterized by GGO with septal thickening, patchy distribution, and aerial bronchogram.

The main CT findings, among 45/60 patients with suspicious CT for COVID-19 infection, were GGOs (40/45, 88.9%) with distribution multifocal and diffuse in 21/40 (52.5%) cases, multifocal/patching in 13/40 (32.5%) (Figure 2 and Figure 3), and monofocal in 6/40 (Figure 4) (15.0%). In 10/45 (22.2%) cases the crazy-paving pattern was verified (Figure 5). Often, the disease was peripheral (13/45, 42.2%). Consolidations were present in 19/45 (33.3%) cases.

## 4. Discussion and Conclusions

Several studies from China have reported that a chest CT scan can reveal suspicious radiological signs for COVID-19 viral infection notwithstanding an RT-PCR negative test result [14]. When present, the findings of COVID-19 on CT (notably peripheral ground-glass opacities) are sensitive but not specific for coronavirus; other pneumonia types resembling COVID-19, particularly viral and *Pneumocystis jirovecii* pneumonia, cryptogenic organizing pneumonia, and acute lung injury from drug toxicity, hypersensitivity, and autoimmune diseases, to name a few pathologies. Moreover, a CT can be normal in early illness, and after each potentially infected patient is scanned, the machine must be completely disinfected. Therefore, CT is not recommended for COVID-19 screening. Although, neither chest CT scans nor X-rays are currently recommended to diagnose COVID-19, CT is used in patients with acute respiratory symptoms to assess the lung disease involvement [7,8,9,10].

Several authors have reported inconsistent results between qualitative RT-PCR and CT findings [26,27]. Tai et al. reported among 1014 enrolled patients that from 60% (34 of 57) to 93% (14 of 15) had initial positive CT scans consistent with COVID-19 before (or parallel to) the initial positive RT-PCR results. Twenty-four of 57 patients (42%) showed improvement on follow-up chest CT scans before the RT-PCR results turned negative [27].

In this manuscript, we assessed the performance of the second reading of the chest CT using a structured report in patients with discordant findings between the RT-PCR test and the first CT diagnosis for COVID-19 viral pneumonia. In a sample of 378 patients with suspicious COVID-19 infection, discordance between RT-PCR and CT findings at first reading was found in 60/378 (15.9%) cases. 

The discrepancy between RT-PCR and CT findings could be linked to the concept that the virus was detected for a medium of 20 days after symptom onset, but infectiousness declined significantly eight days after symptom onset [28]. As reported in [29], a variation in the false-negative rate of RT-PCR during the time of patients infection was demonstrated: over the four days of infection before the typical time of symptom onset (day five), the probability of a false-negative result in an infected person decreased from 100% on day one to 67% on day four. On the day of symptom onset, the median false-negative rate was 38%, this decreased to 20% on day eight (three days after symptom onset) then began to increase again from 21% on day nine to 66% on day 21. Serial testing in symptomatic patients would almost certainly reduce the false-negative rate.

Instead, we evaluated the difference in CT diagnostic performance between the first reading and the second reading with a structured report. The second reading, and then the use of a structured report for COVID-19 diagnosis, changed the CT diagnosis in 16/60 (26.7%) cases contributing to increasing the concordance with the RT-PCR. Among these 60 cases, we reported eight false negatives with positive RT-PCR and 36 false positives with negative RT-PCR. Therefore, despite the sensitivity and the positive predictive value being high in our population with high pretest disease probability, the specificity was low (53.8%); this could be linked to the question that CT radiological patterns for COVID-19 were similar to those of other infections. 

The main CT findings, among 45/60 patients with suspicious CT diagnosis for COVID-19 viral pneumonia, were GGOs with distribution multifocal and diffuse or multifocal/patching. 

Our findings were in accordance with other results reported in the literature. Sensitivities and specificities values of chest CT examination in the identification of COVID-19 viral infection was very variable: from 60% to 98% and from 25% to 53%, respectively [30,31,32,33,34]. This variability could be linked to the study’s retrospective nature. Moreover, chest CT demonstrated a high positive predictive value (92%) to identify COVID-19 disease, the reported negative predictive was low (42%) [31]. Ai et al. [34] testified that the chest CT sensitivity in suspicious COVID-19 patients was 97% considering positive RT-PCR test. Considering all cases with a negative RT-PCR test, 308/413 (75%) of patients had positive chest CT findings. These findings suggest that CT may not be an adequate screening method in the early phases of the COVID-19 disease.

The goal of structured reporting in the setting of COVID-19 pneumonia is to provide a standardized language in the description of the CT findings to decrease reporting variability and allow for the immediacy of the report, reduce waiting times, facilitate the result understanding by other specialists, and reduce the uncertainty in reporting findings potentially attributable to this infection, thereby allowing better integration into clinical decision making. While we do not currently recommend the use of CT screening for COVID-19 pneumonia, we suggest using a standardized language when specifically asked to address whether or not findings of COVID-19 pneumonia may be present on CT images and propose language that could be placed in the radiologist report [25].

The main limitation of the present study is the retrospective and monocentric nature of the study. The use of a qualitative RT-PCR evaluation did not allow for the performance of the curve or expression level of each patient from RT-PCR and the corresponding diagnostic confidence level to define it by chest CT.

Moreover, the study was conducted on a population with a high pretest probability of COVID-19 infection. 

In conclusion, double reading of CT could increase the diagnostic confidence of radiological interpretation in COVID-19 patients in a pandemic area. Using second readers and a structured report for COVID-19 diagnosis could reduce the rate of discrepant cases between RT-PCR result and CT diagnosis for COVID-19 viral pneumonia.

## Figures and Tables

**Table 1:** Demographic characteristics and CT findings and performance in the detection of pneumonia infection by Coronavirus Disease (COVID-19) in patients with confirmed COVID-19 upon an RT-PCR test.

Age (y)	Positive for COVID-19 upon RT-PCR Test		Negative for COVID-19 upon RT-PCR Test		*p*-Value
**Median**	70.0		77.5		>0.05
**Range**	29–93		32–89		
	**Tot**	%	**Tot**	%	***p*** **-value**
**Sex, no. (%) of patients**					
Male	226	68.9	31	62.0	**0.01**
Female	102	31.9	29	58.0	
**CT diagnosis at first CT reading**					
Positive	283	94.3	43	55.1	**<<0.001**
Negative	17	5.7	35	44.9	
**CT diagnosis at second CT reading**					
Positive	292	97.3	36	46.2	**<<0.001**
Negative	8	2.7	42	53.8	
**Cases with concordance between RT-PCR and CT diagnosis**					
Median value of temporal windows between RT-PCR and CT execution	0		0		>0.05
Range of temporal windows between RT-PCR and CT execution	−9–11		−1–4		
**Cases with discordance between RT-PCR and CT diagnosis**					
Median value of temporal windows between RT-PCR and CT execution	0		0		>0.05
Range of temporal windows between RT-PCR and CT execution	−2–4		−8–4		
**CT Findings in 50 patients with discordance between RT-PCR and CT diagnosis after second CT reading**					
Presence of GGOs with consolidation	2		12		**>0.05**
Presence of GGOs without consolidation	5		20		
Presence of consolidation without GGOs	1		4		

Note: *p*-value was evaluated for the continuous variable by the Mann–Whitney test and by the Chi-square test with Yates correction for categorical ones. The *p*-values reported in bold were considered significant.

**Table 2:** CT findings considered for assignment of the diagnostic confidence level.

Diagnostic Confidence Level	CT Characteristics
High diagnostic confidence level	Bilateral multifocal GGO with predominantly peripheral distribution associated or not with septal thickening (crazy paving) and/or consolidations; multifocal GGO of rounded morphology associated or not with crazy paving and/or consolidations; multifocal GGO associated with findings of organizing pneumonia.
Intermediate diagnostic confidence level	GGO with diffuse distribution associated or not with crazy paving and/or consolidations; bilateral multifocal GGO and/or consolidations without a prevalent peripheral distribution and without rounded morphology; unilateral GGO with or without consolidation.
Low diagnostic confidence level	Isolated small areas of GGO and/or consolidations with non-rounded morphology were included in the low confidence level.
Negative for COVID-19	Cases without the described alterations and with one or more of the following alterations were considered indicative of other diagnoses: isolated lobar or segmental consolidations, presence of solid or caveated nodules, presence of micro-nodules (centro-lobular micro-nodules and “tree in bud” pattern), smooth thickening of the interlobular septa with pleural effusion.

## Data Availability

No additional data are available.
